# Subcellular Compartmentalization and Chemical Forms of Lead Participate in Lead Tolerance of *Robinia pseudoacacia* L. with *Funneliformis mosseae*

**DOI:** 10.3389/fpls.2017.00517

**Published:** 2017-04-10

**Authors:** Li Huang, Haoqiang Zhang, Yingying Song, Yurong Yang, Hui Chen, Ming Tang

**Affiliations:** ^1^College of Life Sciences, Northwest A&F UniversityYangling, China; ^2^College of Forestry, Northwest A&F UniversityYangling, China

**Keywords:** arbuscular mycorrhizal fungus, symbiosis, bioremediation, Pb, plant tolerance

## Abstract

The effect of arbuscular mycorrhizal fungus on the subcellular compartmentalization and chemical forms of lead (Pb) in Pb tolerance plants was assessed in a pot experiment in greenhouse conditions. We measured root colonization, plant growth, photosynthesis, subcellular compartmentalization and chemical forms of Pb in black locust (*Robinia pseudoacacia* L.) seedlings inoculated with *Funneliformis mosseae* isolate (BGC XJ01A) under a range of Pb treatments (0, 90, 900, and 3000 mg Pb kg^-1^ soil). The majority of Pb was retained in the roots of *R. pseudoacacia* under Pb stress, with a significantly higher retention in the inoculated seedlings. *F. mosseae* inoculation significantly increased the proportion of Pb in the cell wall and soluble fractions and decreased the proportion of Pb in the organelle fraction of roots, stems, and leaves, with the largest proportion of Pb segregated in the cell wall fraction. *F. mosseae* inoculation increased the proportion of inactive Pb (especially pectate- and protein-integrated Pb and Pb phosphate) and reduced the proportion of water-soluble Pb in the roots, stems, and leaves. The subcellular compartmentalization of Pb in different chemical forms was highly correlated with improved plant biomass, height, and photosynthesis in the inoculated seedlings. This study indicates that *F. mosseae* could improve Pb tolerance in *R. pseudoacacia* seedlings growing in Pb polluted soils.

## Introduction

Lead (Pb) is one of the most widespread toxic metals (Fahr et al., 2013) and tends to remain in soil for long periods of time (LaBelle et al., 1987). Pb pollution may be naturally occurring or result from anthropogenic activities (Yadav, 2010), creating potential hazards to ecosystems (Lantzy and Mackenzie, 1979). Pb pollution has also been a serious threat to human health and food safety (Cuypers et al., 2016), especially harmful to young children (Datko-Williams et al., 2014). Over the last decade, evidence has been shown that Pb exposure-related health injury occur already at lower blood Pb levels than previously documented (Henry et al., 2015). Recently, the Centers for Disease Control and Prevention has lowered the reference value of blood Pb level to 5 μg dL^-1^ (Burns and Gerstenberger, 2014). To adhere the Pb reference value the diminishment of Pb level in soils is of high interest at present. As the demand for conversion of post-industrial lands continues to raise the remediation of Pb polluted soils represents one principal task to fulfill this requirement (Yousaf et al., 2016).

The established traditional techniques (e.g., washing, electrochemical methods, thermal processes, physical separation, stabilization/solidification, and burial) for clean-up of metal contaminated soils are generally expensive and harmful to soil microbial diversity (Rajkumar et al., 2012). Bioremediation has received increasing attention as it represents an eco-friendly and low-cost technology (Kotrba et al., 2009) using living plants (Janoušková and Pavlíková, 2010) or microorganisms (Brito et al., 2014) to remove toxic heavy metals (HM) in contaminated areas. Various plants growing in HM contaminated soils have developed specialized mechanisms for tolerating intracellular HM. Subcellular compartmentalization is an important way to eliminate the toxicity of HM in plants (Cobbett, 2003). Once taken up by plants, HM can exist in different chemical forms, including inorganic, water-soluble, pectate- and protein-integrated, undissolved phosphate and oxalate forms (Wu et al., 2005). For example, conversion of cadmium (Cd) into insoluble phosphate precipitates and pectate- or protein-bound forms is the primary means for reducing Cd mobility and toxicity in *Nasturtium officinale* (Wang J.B. et al., 2015). Copper (Cu) bound to the cell walls in fibrous roots of *Malus sieversii* mainly exists in phosphate and oxalate forms, which can explain some of the variation in Cu sensitivity in *M. sieversii* (Wang et al., 2016). Recently, Li et al. (2016) reported that the cell walls and intercellular spaces are the main location of Pb accumulation in the roots of *Conyza canadensis*. The cell walls restrict Pb uptake into plant roots and act as an important barrier to protect root cells (Wang Y. et al., 2015). Pb fixation by pectates and proteins in the cell walls and sequestration in the vacuoles were found to be responsible for Pb detoxification in *Lolium perenne* (He et al., 2015).

Arbuscular mycorrhizal fungi (AMF) are ubiquitous in terrestrial ecosystems (Sędzielewska-Toro and Delaux, 2016), forming symbiotic interactions with 80% of land plants. AMF can improve plant nutrient acquisition (Orrell and Bennett, 2013), enhance plant photosynthesis, and influence the fate of HM in both plants and the soil (Brito et al., 2014; Saia et al., 2015). Inoculation with *Funneliformis mosseae* resulted in a higher Pb tolerance of *Eucalyptus grandis* × *urophylla* and has been related to the retention of Pb in the roots, the binding of Pb to the cell walls, vacuolar compartmentalization of Pb in the soluble fraction, and increase in the proportion of less bioactive Pb (Liao et al., 2014). Thus, AMF may be used to advance plant-based environmental remediation by altering subcellular compartmentalization and chemical forms of HM in plants. Hence, it is necessary to select an appropriate HM-tolerant plants species that could form a symbiotic relationship with AMF.

Black locust (*Robinia pseudoacacia* L.) is a leguminous tree species widely planted on the Loess Plateau, China (Zhang et al., 2016). *R. pseudoacacia* is frequently found in HM contaminated areas and it may serve as an indicator of Pb pollution (Serbula et al., 2012). *R. pseudoacacia* plants are well grown and commonly colonized by AMF such as *F. mosseae* in the Qiandongshan lead–zinc polluted area (Yang et al., 2015b,c). Recent studies have shown that photosynthesis and antioxidant enzymes (e.g., superoxide dismutase and ascorbate peroxidases) in the leaves of *R. pseudoacacia* are enhanced by *F. mosseae* under Pb stress (Yang et al., 2015a). Additionally, *F. mosseae* is effective at accumulating Pb in plant root systems (Yang et al., 2015a). However, detailed information on the impact of *F. mosseae* on the subcellular compartmentalization and chemical forms of Pb in *R. pseudoacacia* under Pb stress are rare. To understand the efficiency of bioremediation of Pb in soils, there is a need to study the molecular mechanism how AMF colonization enhances Pb tolerance in woody legumes (Leguminosae) such as *R. pseudoacacia*.

One objective of our study was aimed to clarify whether *F. mosseae* is of significance for Pb tolerance in seedlings of *R. pseudoacacia*. For this purpose, inoculated as well as non-inoculated seedlings with *F. mosseae* were exposed to different Pb levels (0, 90, 900, and 3000 mg Pb kg^-1^ soil). The impact of Pb was studied on the basis of plant growth, gas exchange (CO_2_ assimilation, stomatal conductance for water vapor) and chlorophyll fluorescence. A second objective of this study was to give detailed insights in the detoxification mechanism of Pb from the molecular perspective, such as subcellular compartmentalization and conversion of Pb into inactive forms, and their alteration due to inoculation with *F. mosseae*. In this respect, the impact of *F. mosseae* on Pb tolerance was studied on the basis of Pb uptake in relation to the proportion of Pb located in different plant tissue, subcellular fractions and chemical forms.

## Materials and Methods

### Fungal Inoculum and Plant

*Funneliformis mosseae* (BGC XJ01A) spores were purchased from the Institute of Plant Nutrition and Resource, Beijing Academy of Agriculture and Forestry Sciences (Beijing, China). The fungus was propagated with fine sand for 3 months using *Zea mays* under greenhouse condition. The average colonization was 91.7%. The fungal inoculum consisted of sand, infected root fragments, external hyphae, and spores (∼26 spores g^-1^).

*Robinia pseudoacacia* L. is a Pb-tolerant tree species (Yang et al., 2015c). The seeds were collected from Northwest A&F University (Yangling, Shaanxi Province, China) in 2013. Seeds were surface sterilized with 10% H_2_O_2_ for 10 min, washed with distilled water for several times, and then soaked for 24 h before germinating on sterile filter paper in a Petri dish in an incubator at 28°C.

### Growth Substrate

Soil used in this study was collected from the surface horizon (0–30 cm) on the campus of Northwest A&F University. The soil was air-dried, homogenized, and ground in a ceramic mill and passed through a 2 mm sieve before performing chemical analyses. After being mixed with washed fine sand (<2 mm), the substrate (sand/soil, 1:2 v/v) was autoclaved at 121°C for 2 h.

The properties of the soil were as follows (per kilogram of dry soil) after autoclaving: pH 7.66 (soil/water = 1:2.5, w/v), organic matter 14.85 g, ammonium-nitrogen 7.37 mg, nitrate-nitrogen 25.77 mg, available phosphorus 11.48 mg, and available potassium 128.96 mg, total Pb 6.58 mg. Measurements were performed according to the method described by Bao (2000).

The autoclaved substrate was divided into four subsamples. To three of the subsamples, Pb(NO_3_)_2_(aq) was added to produce substrates with different Pb levels, i.e., 90, 900, and 3000 mg kg^-1^ (mass of Pb/mass of dry soil) based on our pre-experiment (Yang et al., 2015c). An equivalent amount of distilled water was added to the control (0 mg Pb kg^-1^ soil). The four subsamples were supplied with an appropriate amount of NH_4_NO_3_ to compensate for the quantity of nitrate added as Pb(NO_3_)_2_. After addition of Pb solution, the growth substrate was allowed to stabilize for 1 month before used.

### Experimental Design and Plant Culture

The experiment was setup as a 4 × 2 factorial design consisting of four Pb levels and one AMF inoculum and non-inoculated control which were arranged in a completely randomized design with 30 replicates per treatment combination. The experiment was performed from March to July 2014 in a greenhouse located at Northwest A&F University. Plants were kept at average room temperature (35/20°C, day/night) under a natural light regime during the period of plant growth. Soil moisture was determined by a soil moisture meter (Field Scout TDR 100, Spectrum Technologies Inc., Plainfield, IL, USA) and maintained at approximately 60% of field capacity by adding the amount of lost water to each pot daily.

Four uniform pre-germinated seeds were sown in each plastic pot (10 cm × 8 cm) with approximately 450 g growth substrate; each pot received 20 g fresh inoculum for mycorrhizal treatment or 20 g sterilized inoculum with 10 mL AMF-free filtrate (10 μm pore size) of unsterilized inoculum (soil:water = 1:10 w/v) as the non-mycorrhizal treatment (Sheng et al., 2008). The seedlings were thinned to one plant per pot 10 days after emergence. Plants were watered daily with tap water (35 mL) and supplemented with 0.25 × fresh Hoagland’s nutrient solution (35 mL) (Hoagland and Arnon, 1950) once a week throughout the growth stage.

After 4 months growth, the photosynthetic parameters and growth of six randomly selected plants per treatment were measured. The whole plants were washed with tap water to remove soil or dust deposits, and the roots were immersed in 20 mM Na_2_EDTA for 15 min to remove metal ions adhering to root surface (Chen et al., 2014). The plants were then washed with deionized water and dried with paper towels. Thereafter, the plants were separated into roots, stems, and leaves. The fresh roots (except for fine roots used for determination of AMF colonization), stems, and leaves were immediately frozen in liquid nitrogen (-196°C) and stored at -70°C for further analysis.

### Physiological Measurements

#### Mycorrhizal Colonization and Growth Parameters

Mycorrhizal colonization (MC) was determined for fresh roots using the method described by Phillips and Hayman (1970). Then the MC was calculated according to Ban et al. (2015). Plant height and stem diameter (at 1 cm above the soil surface) were measured by precision straight edge (Deli 8200, Ningbo, China) and vernier caliper (Yifante ECV150C, Wuxi, China), respectively. Root dry weight, stem dry weight, and leaf dry weight were recorded after oven-drying to constant weight at 70°C. Total biomass was calculated as: root dry weight + stem dry weight + leaf dry weight.

#### Gas Exchange and Chlorophyll Fluorescence Parameters

Six healthy and functional leaves (youngest fully expanded from six different plants) were taken from each treatment and each leaf was measured five times for every photosynthetic parameter (Huang et al., 2011). Net CO_2_ assimilation rate (*A*), stomatal conductance to water vapor (gsw), intercellular CO_2_ concentration (C_i_), and transpiration (*E*) were measured with a portable open-flow gas exchange system LI-6400 (LI-COR, USA) on a cloudless day from 9:00 to 11:30 a.m. in the glasshouse at 25°C. Automatic measurements were made under optimal conditions: photosynthetically active radiation 1000 ± 12 μmol m^-2^ s^-1^, CO_2_ concentration 350 ± 2 cm^3^ m^-3^, leaf temperature 28.0 ± 0.8°C, relative humidity 60%, ambient water vapor pressure 1.35 kPa, and flow rate of atmosphere 0.5 dm^3^ min^-1^.

Fluorescence assays were addressed to the same leaves as used for the photosynthetic measurements. The fluorescence parameters were measured at room temperature between 9:00 and 11:30 a.m. by a MINI-Imaging-PAM system (Imaging-PAM, Heinz Walz GmbH, Germany) as described by Gong et al. (2013). The seedlings were placed in darkness for 30 min and the minimal fluorescence in the dark-adapted state (F_o_) was recorded. A saturating pulse of irradiation (2000 μmol m^-2^ s^-1^, 3 s) was applied to determine the maximal fluorescence (F_m_) in the dark-adapted state. Then, the leaves were illuminated with actinic light (300 μmol m^-2^ s^-1^, 10 min) for evaluating the minimal fluorescence (F_o_′) and maximal fluorescence (F_m_′). The incident photosynthetically active irradiance (EPAR), effective photochemical efficiency of PSII (ΦPSII), and steady-state value of fluorescence (F_s_) under actinic light were recorded. Using both light and dark fluorescence parameters, we calculated: (1) the maximal quantum yield of PSII in the dark-adapted state, F_v_/F_m_ = (F_m_ - F_o_)/F_m_, (2) the effective photochemical efficiency of PSII, ΦPSII = (F_m_′ - F_s_)/F_m_′, (3) the potential activity of PSII, F_v_/F_o_ = (F_m_ - F_o_)/F_o_, (4) the photosynthetic electron transport rate, ETR = (F_m_′ - F_s_)/F_m_′ × EPAR, (5) the photochemical quenching coefficient, qP = (F_m_′ - F_s_)/(F_m_′ - F_o_′), and (6) the non-photochemical quenching coefficient, qN = (F_m_ - F_m_′)/F_m_′.

#### Pb Analysis

To determine the fractions of Pb present in *R. pseudoacacia*, a fractionation procedure was adapted from a published protocol with slight modifications (Weigel and Jäger, 1980). Cells were separated by gradient centrifugation at 4°C into three different fractions: cell wall fraction (FI), organelle fraction (FII), and soluble fraction (FIII). In brief, 0.5 g of frozen tissues was homogenized in a pre-cold (4°C) extraction buffer [containing 50 mM Tris–HCl (pH 7.5), 250 mM sucrose, and 1.0 mM dithiothreitol (C_4_H_10_O_2_S_2_)] at the ratio of 1:10 (w/v) with a chilled mortar and a pestle. The homogenate was passed through a nylon cloth (80 μm mesh size) and liquid was squeezed from the residue. The residue on the cloth was washed twice with a homogenization buffer. The pooled washes, together with the first filtrate, were centrifuged at 300 × *g* for 30 s. The resulting pellet combined with the residue of nylon cloth filtration was designated as FI, containing mainly of cell walls and cell wall debris. The resulting supernatant solution was further centrifuged at 12,000 × *g* for 45 min. The pellet and supernatant solution were referred to as FII and FIII, respectively. The different fractions were dried at 70°C on an electric heating plate to a volume of approximately 1–2 mL and then wet-digested with concentrated acid HNO_3_/HClO_4_ (4:1, v/v). The recovery rate of Pb was calculated as: (cell wall fraction + organelle fraction + soluble fraction) Pb/total Pb × 100%. The proportion of Pb of the fraction was calculated with the following formula: Pb content in each fraction/total Pb content in the respective tissue × 100%.

To detect different chemical forms of Pb in plant tissues, we performed five extraction processes using for each a different extraction solvent (Wang et al., 2008), i.e., 80% ethanol (*F*_Ethanol_; inorganic, soluble Pb), distilled water (*F*_d-H_2_O_; organic, soluble Pb), 1 M sodium chloride (*F*_NaCl_; pectate and protein- Pb), 2% acetic acid (*F*_HOAc_; Pb phosphate), 0.6 M hydrochloric acid (*F*_HCl_; Pb oxalate), and Pb in residues (*F*_Residue_). Briefly, 0.5 g of frozen tissues were homogenized with a mortar and a pestle in liquid nitrogen (–196°C), diluted at a ratio of 1:50 (w/v) with the extraction solution, and then shaken for 22 h at 25°C. The resulting extracts were centrifuged at 5000 × *g* for 10 min. The precipitates were washed twice by re-suspending in the respective extraction medium, shaking at 25°C for 2 h, and centrifuging at 5000 × *g* for 10 min. The supernatants from each of the three repetitions were then pooled for each of the five extraction solutions. The resulting supernatant solvent from extraction solutions were evaporated on an electric heating plate to a volume of approximately 1–2 mL, followed by digestion with HNO_3_:HClO_4_ (4:1, v/v). To measure the Pb content in residues, plant materials were digested with HNO_3_–HClO_4_ (4:1, v/v) at the end of the sequential extraction. The recovery rate of Pb was calculated as: (*F*_Ethanol_ + *F*_H_2_O_ + *F*_NaCl_ + *F*_HOAc_ + *F*_HCl_ + *F*_Residue_) Pb/total Pb × 100%. The proportion of Pb of the chemical form was calculated as: Pb content of the chemical form/total Pb content in the respective tissue × 100%.

To determine the total Pb content in different tissues, oven-dried subsamples were ground into powder and sieved through a nylon mesh (100 μm). Then, 0.5 g aliquots of the samples were wet-digested in HNO_3_:HClO_4_ (4:1, v:v). Total Pb content as well as Pb content in different subcellular fractions and chemical forms was determined by atomic absorption spectrophotometry (Shimadzu AA-6300C, Kyoto, Japan). The flame composition was acetylene (flow rate 2 L min^-1^) and air (flow rate 15 L min^-1^). The working conditions of the instrument were as follows: current 7.5 mA, wavelength 217 nm, slit width 1.3 nm, burner height 7.5 mm, negative high voltage of photomultiplier tube 576 V, and auxiliary gas pressure 160 kPa.

Quality assurance and quality control for Pb in *R. pseudoacacia* was conducted using the standard reference material bush leaves (GBW07602), which was treated in the same way as the plant samples. The recovery for standard was approximately 95.3–108.5%. The standard solutions with different concentrations in the range of 1–10 μg mL^-1^ were aspirated in turn into flame and their absorbance values were recorded at 217 nm wavelength. The calibration curve was constructed by plotting on a linear graph paper using the absorbance of standards versus their concentrations. The correlation equations were defined as *y* = 0.019*x* + 0.004, *R*^2^ = 0.999. Reagent blanks and analytical duplicates were also used where appropriate, to ensure the accuracy and precision in the analysis. Pb content was determined three times for each sample and the relative standard deviation (SD) of Pb content was calculated (<4.0%).

### Statistical Analysis

All values were expressed as mean ± SD (*n* = 6). The Kolmogorov–Smirnov test was applied to assess data normality and the Levene test for homogeneity of variance in SPSS 22.0 (SPSS Inc., Chicago, IL, USA). All the original datasets followed a normal distribution in this study. Potential differences among different Pb treatments were analyzed using two-way and three-way analysis of variance (ANOVA) followed by Duncan’s multiple comparison at *P* < 0.05. An independent *t*-test was performed to detect significant differences in plant growth and physiological parameters between the inoculated and non-inoculated controls within one Pb level. A two-factorial ANOVA and Pearson correlation analysis were performed to examine the influence of Pb exposure as well as the mycorrhizal treatment on physiological parameters as well as on the proportions of Pb in subcellular fractions and chemical forms and to detect possible correlations between these traits (*n* = 48). Graphs were drawn using SigmaPlot 10.0 (Systat Software, San Jose, CA, USA).

## Results

### Mycorrhizal Colonization and Plant Growth

**FIGURE 1:**
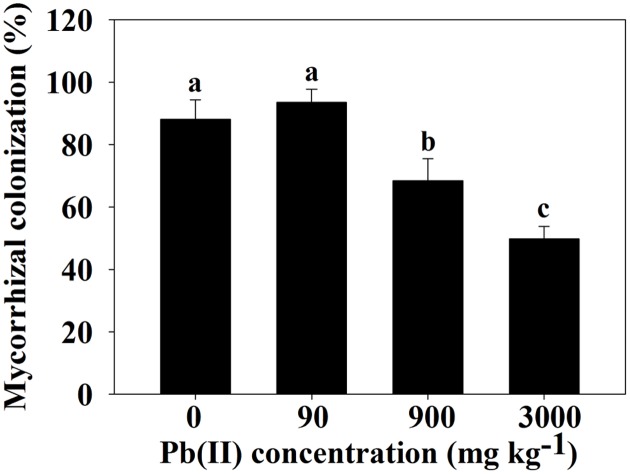
**Colonization of *Robinia pseudoacacia* roots by *Funneliformis mosseae* in response to 0, 90, 900, and 3000 mg Pb kg^-1^ soil for 4 months**. Shown are means ± SD (*n* = 6). Different lower case letters indicate significant differences among different Pb levels within inoculated treatment (*P* < 0.05; ANOVA with *post hoc* Duncan).

None of the plants from non-inoculated treatments were colonized by *F. mosseae*. The symbiotic relationship between *F. mosseae* and *R. pseudoacacia* was well established in inoculated treatments irrespective of Pb treatment. Inoculated *R. pseudoacacia* showed 88.16% MC under control condition (0 mg Pb kg^-1^ soil), while low Pb treatment (90 mg Pb kg^-1^ soil) resulted in a slight increase in MC (93.56%). With increasing Pb level, MC of *F. mosseae* was significantly decreased and the lowest MC (49.77%) appeared at 3000 mg Pb kg^-1^ soil (**Figure 1**).

**FIGURE 2:**
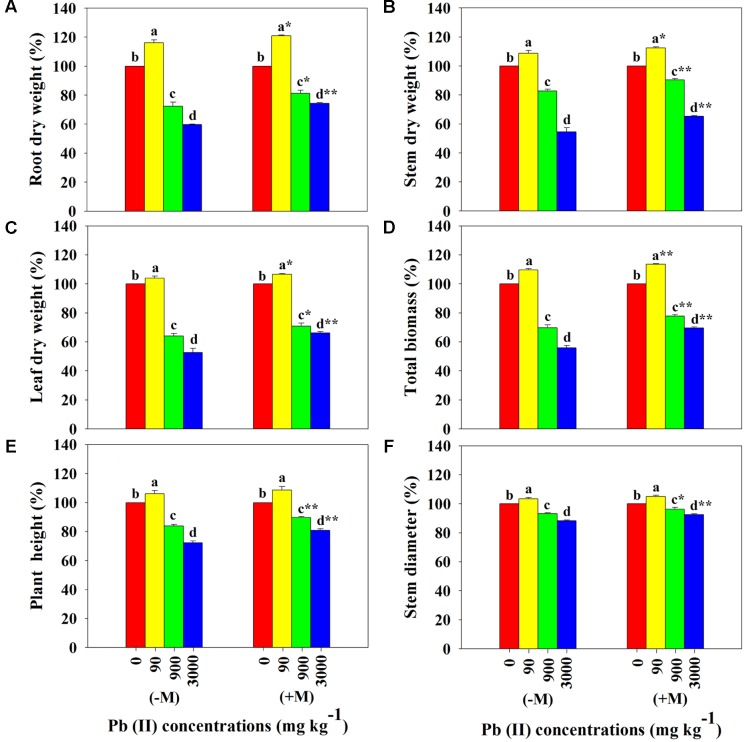
**Relative differences in plant growth of *Robinia pseudoacacia* seedlings with (+M) or without (–M) *Funneliformis mosseae* in response to 0, 90, 900, and 3000 mg Pb kg^-1^ soil for 4 months**. **(A)** Root dry weight, **(B)** stem dry weight, **(C)** leaf dry weight, **(D)** total biomass, **(E)** plant height, and **(F)** stem diameter. Shown are means ± SD (*n* = 6). Asterisks indicate significant differences between inoculated and non-inoculated seedlings within one Pb level (^∗^*P* < 0.05, ^∗∗^*P* < 0.01; *t*-test). Different lower case letters indicate significant differences among different Pb levels within inoculated or non-inoculated treatment (*P* < 0.05; ANOVA with *post hoc* Duncan).

Under control conditions (0 mg Pb kg^-1^ soil), the growth parameters of *R. pseudoacacia* seedlings showed significant differences between non-inoculated and inoculated treatments. Inoculated seedlings showed significantly higher plant biomass (roots, stems, leaves, and total), plant height, and stem diameter than non-inoculated seedlings (**Table 1**). Compared to the controls, seedlings exposed to the lowest Pb level (90 mg Pb kg^-1^ soil) showed a significant improvement in plant growth, which was greater for inoculated seedlings than for non-inoculated seedlings (e.g., by 21% versus 16% in root dry weight). In contrast, exposure to higher Pb levels (900 and 3000 mg Pb kg^-1^ soil) resulted in a significant reduction in plant growth, which was greater for non-inoculated seedlings than for inoculated seedlings (e.g., by 41% versus 26% in root dry weight at 3000 mg Pb kg^-1^ soil; **Figure 2**).

**Table 1:** Plant growth and physiological parameters of *Robinia pseudoacacia* seedlings with (+M) or without (-M) *Funneliformis mosseae* at 0 mg Pb kg^-1^ soil for 4 months.

Parameter	-M	+M
MC (%)	0	88.16 ± 6.18^∗∗∗^
Root dry weight (g plant^-1^)	1.99 ± 0.03	2.27 ± 0.06^∗∗^
Stem dry weight (g plant^-1^)	0.58 ± 0.02	0.66 ± 0.02^∗∗^
Leaf dry weight (g plant^-1^)	2.16 ± 0.03	2.33 ± 0.02^∗∗∗^
Total biomass (g plant^-1^)	4.73 ± 0.08	5.26 ± 0.06^∗∗∗^
Plant height (cm)	25.03 ± 0.86	42.20 ± 0.56^∗∗∗^
Stem diameter (mm)	2.95 ± 0.03	3.35 ± 0.03^∗∗∗^
A (μmol CO_2_ m^-2^ s^-1^)	6.53 ± 0.45	8.39 ± 1.12^∗∗^
gsw (mmol H_2_O m^-2^ s^-1^)	0.11 ± 0.00	0.14 ± 0.00^∗∗∗^
C_i_ (μmol CO_2_ mol^-1^)	150.67 ± 5.25	144.73 ± 4.99 ^NS^
E (mmol H_2_O m^-2^ s^-1^)	2.61 ± 0.15	2.90 ± 0.09^∗^
F_v_/F_m_	0.74 ± 0.01	0.83 ± 0.01^∗∗∗^
ΦPSII	0.72 ± 0.01	0.80 ± 0.01^∗∗∗^
F_v_/F_o_	2.89 ± 0.19	4.91 ± 0.28^∗∗∗^
ETR	31.52 ± 1.99	39.19 ± 0.52^∗∗^
qP	0.79 ± 0.01	0.85 ± 0.01^∗∗∗^
qN	0.68 ± 0.01	0.58 ± 0.01^∗∗∗^

*Shown are means ± SD (*n* = 6). Asterisks indicate significant differences between inoculated and non-inoculated seedlings at 0 mg Pb kg^-*1*^ soil (^∗^*P* < 0.05, ^∗∗^*P* < 0.01, ^∗∗∗^*P* < 0.001; *t*-test). NS, not significant; MC, mycorrhizal colonization; *A*, net CO_2_ assimilation rate; gsw, stomatal conductance to water vapor; C_i_, intercellular CO_*2*_ concentration; *E*, transpiration rate; *F*_*v*_/*F*_*m*_, maximum quantum yield in the dark-adapted state of PSII; *Φ*PSII, actual quantum yield of PSII in light-adapted steady state; *F*_*v*_/*F*_*o*_, potential activity of PSII; ETR, electron transport rate; qP, photochemical quenching coefficient; qN, non-photochemical quenching values*.

### Gas Exchange and Chlorophyll Fluorescence

**FIGURE 3:**
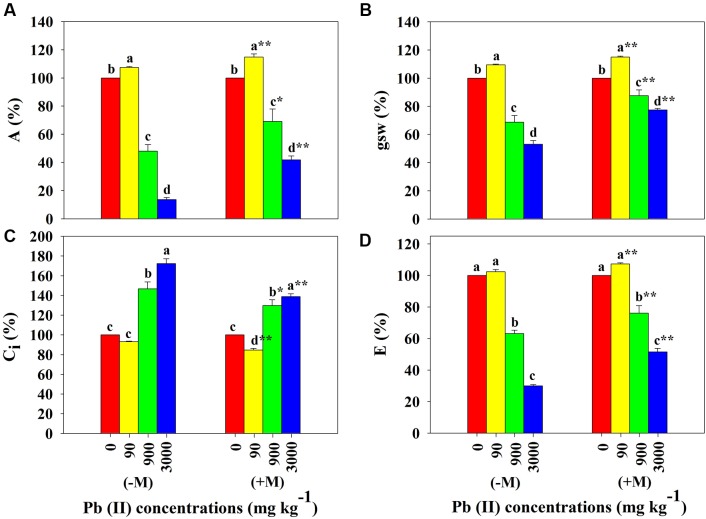
**Relative differences in gas exchange in leaves of *Robinia pseudoacacia* seedlings with (+M) or without (–M) *Funneliformis mosseae* in response to 0, 90, 900, and 3000 mg Pb kg^-1^ soil for 4 months**. **(A)** Net CO_2_ assimilation rate, A; **(B)** stomatal conductance to water vapor, gsw; **(C)** intercellular CO_2_ concentration, C_i_; and **(D)** transpiration rate, E. Shown are means ± SD (*n* = 6). Asterisks indicate significant differences between inoculated and non-inoculated seedlings within one Pb level (^∗^*P* < 0.05, ^∗∗^*P* < 0.01; *t*-test). Different lower case letters indicate significant differences among different Pb levels within inoculated or non-inoculated treatment (*P* < 0.05; ANOVA with *post hoc* Duncan).

In the leaves of control seedlings at 0 mg Pb kg^-1^ soil, gas exchange parameters (except for C_i_) significantly differed between non-inoculated and inoculated treatments. Inoculated seedlings had significantly higher A, gsw, and E values compared to non-inoculated seedlings (**Table 1**). Compared to control data, A, gsw, and E were significantly increased at 90 mg Pb kg^-1^ soil and significantly reduced at 900 and 3000 mg Pb kg^-1^ soil in both inoculated and non-inoculated seedlings. However, the C_i_ values of both inoculated and non-inoculated seedlings were higher at 900 and 3000 mg Pb kg^-1^ soil compared to the respective seedlings at lower Pb level. Moreover, inoculated seedlings had significantly higher A, gsw, and E and lower C_i_ compared to non-inoculated seedlings at the highest Pb level (e.g., by 58, 22, 44, and 39% versus 86, 47, 71, and 72%, respectively, at 3000 mg Pb kg^-1^ soil; **Figure 3**).

**FIGURE 4:**
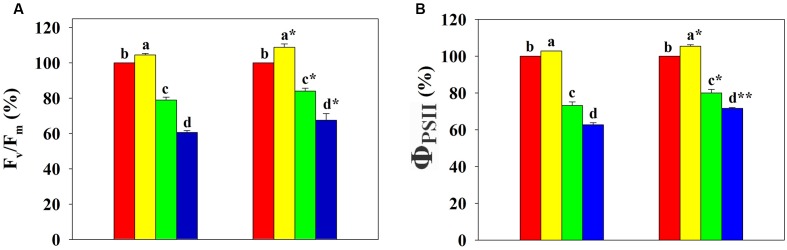
**Relative differences in chlorophyll fluorescence in leaves of *Robinia pseudoacacia* seedlings with (+M) or without (–M) *Funneliformis mosseae* inoculation in response to 0, 90, 900, and 3000 mg Pb kg^-1^ soil for 4 months**. **(A)** The maximum quantum yield in the dark-adapted state of PSII, F_v_/F_m_; and **(B)** the actual quantum yield of PSII in light-adapted steady state, ΦPSII. Shown are means ± SD (*n* = 6). Asterisks indicate significant differences between inoculated and non-inoculated seedlings within one Pb level (^∗^*P* < 0.05, ^∗∗^*P* < 0.01; *t*-test). Different lower case letters indicate significant differences among different Pb levels within inoculated or non-inoculated treatment (*P* < 0.05; ANOVA with *post hoc* Duncan).

Under control conditions, we observed significantly higher F_v_/F_m_, ΦPSII, F_v_/F_o_, ETR, and qP, and significantly lower qN in the leaves of inoculated seedlings compared to non-inoculated seedlings (**Table 1**). Compared to control levels, F_v_/F_m_, ΦPSII, F_v_/F_o_, ETR, and qP of both inoculated and non-inoculated seedlings increased at 90 mg Pb kg^-1^ soil, followed by a significant reduction at 900 and 3000 mg Pb kg^-1^ soil. On the contrary, the qN showed a decrease at 90 mg Pb kg^-1^ soil, while an increase was found at 900 and 3000 mg Pb kg^-1^ soil. Inoculated seedlings showed significantly higher F_v_/F_m_, ΦPSII, F_v_/F_o_, ETR, and qP and significantly lower qN compared to non-inoculated seedlings at the highest Pb level (e.g., by 33, 28, 43, 31, 32, and 14% versus 39, 37, 75, 41, 39, and 28%, respectively, at 3000 mg Pb kg^-1^ soil; **Figure 4** and Supplementary Figure S1).

### Pb Uptake and Translocation

Under control conditions, no significant difference in Pb content between non-inoculated and inoculated treatments was detectable. Compared to the control conditions, exposure to Pb resulted in an increase in Pb content in all tissues for inoculated and non-inoculated seedlings in a concentration-dependent manner, with the highest Pb content found in the roots under all Pb treatments. Inoculated seedlings showed significantly higher Pb content in the roots and stems at 90, 900, and 3000 mg Pb kg^-1^ soil and significantly lower Pb content in the leaves at 3000 mg Pb kg^-1^ soil compared to non-inoculated seedlings under Pb exposure (**Table 2**).

**Table 2:** Pb content in roots, stems, and leaves of *Robinia pseudoacacia* seedlings with (+M) or without (-M) *Funneliformis mosseae* exposed to 0, 90, 900, and 3000 mg Pb kg^-1^ soil for 4 months.

	Pb content in plant tissues (μg plant^-1^)		
Pb treatment (mg Pb kg^-1^ soil)	Root	Stem	Leaf
**0**			
-M	1.17 ± 0.05g	0.14 ± 0.00g	0.31 ± 0.01d
+M	1.23 ± 0.02g	0.11 ± 0.00g	0.26 ± 0.01d
**90**			
-M	178.66 ± 4.88f	16.78 ± 0.49f	38.07 ± 0.68b
+M	270.17 ± 6.88e	20.32 ± 0.56e	34.58 ± 2.22b
**900**			
-M	335.75 ± 52.45d	36.70 ± 2.19d	65.95 ± 2.91c
+M	882.26 ± 27.25b	67.62 ± 2.77b	63.08 ± 2.96c
**3000**			
-M	758.06 ± 35.04c	47.16 ± 2.23c	112.77 ± 7.77a
+M	1342.12 ± 60.19a	73.88 ± 2.85a	94.71 ± 1.17b

*Shown are means ± SD (*n* = 6). Different lower case letters in a column indicate significant differences between inoculated and non-inoculated seedlings within the same tissue at different Pb levels (*P* < 0.05; ANOVA with *post hoc* Duncan)*.

### Subcellular Compartmentalization of Pb

**FIGURE 5:**
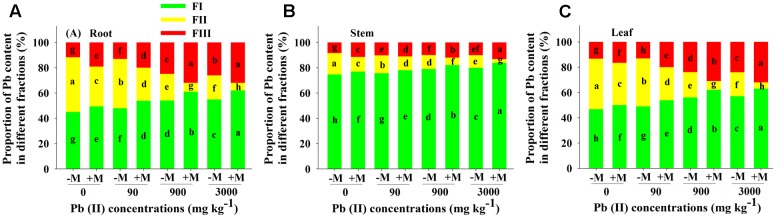
**Proportion of Pb in different subcellular fractions in roots. (A)**, stems **(B)**, and leaves **(C)** of *Robinia pseudoacacia* seedlings with (+M) or without (–M) *Funneliformis mosseae* in response to 0, 90, 900, and 3000 mg Pb kg^-1^ soil for 4 months. Shown are means ± SD (*n* = 6). Different lower case letters within a column indicate significant differences between inoculated and non-inoculated seedlings within the same tissue among different Pb levels (*P* < 0.05; ANOVA with *post hoc* Duncan). Cells were separated by gradient centrifugation at 4°C into cell wall (FI), organelle (FII), and soluble (FIII) fractions.

The recovery rate of Pb was higher than 90% for most treatments. The proportion of Pb content in each subcellular fraction was related to the total Pb content accumulated in the respective tissue. Irrespective of Pb treatment or AMF inoculation, the proportion of Pb was highest in the FI fraction, ranging from 49 to 84% (**Figure 5**). Under control conditions, the proportions of Pb in the subcellular fractions of each tissue significantly differed between non-inoculated and inoculated seedlings. Both the FI and FIII were significantly higher and the FII was lower for inoculated seedlings compared to non-inoculated seedlings. Compared to control conditions, exposure to Pb (90, 900, and 3000 mg Pb kg^-1^ soil) resulted in a significant increase in the proportions of FI and FIII in all analyzed tissues in a concentration-dependent manner, and both fractions were significantly higher for inoculated seedlings than for non-inoculated seedlings (e.g., 62% versus 55% for FI in roots of seedlings at 90 mg Pb kg^-1^ soil). In contrast, the proportion of FII was significantly decreased in the tissues of seedlings under Pb exposure, and this fraction was significantly lower for inoculated seedlings compared to non-inoculated seedlings (e.g., 26% versus 39% in roots of seedlings at 90 mg Pb kg^-1^ soil; **Figure 5**).

### Chemical Forms of Pb

**FIGURE 6:**
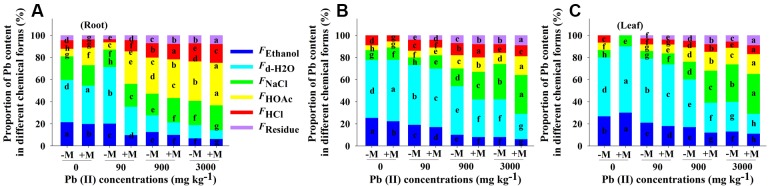
**Proportion of Pb in different chemical forms in roots (A)**, stems **(B)**, and leaves **(C)** of *Robinia pseudoacacia* seedlings with (+M) or without (–M) *Funneliformis mosseae* in response to 0, 90, 900, and 3000 mg Pb kg^-1^ soil for 4 months. Shown are means ± SD (*n* = 6). Different lower case letters within a column indicate significant differences between inoculated and non-inoculated seedlings within the same tissue among different Pb levels (*P* < 0.05; ANOVA with *post hoc* Duncan). Different chemical forms of Pb were sequentially extracted by 80% ethanol (*F*_Ethanol_), deionized H2O *F*_d-H_2_O_), 1 M NaCl (*F*_NaCl_), 2% HOAc (*F*_HOAc_), and 0.6 M HCl (*F*_HCl_). *F*_Residue_, Pb in residues.

The proportion of Pb content in each of chemical form was also related to the total Pb content accumulated in the respective tissue (**Figure 6**). For control seedlings (0 mg Pb kg^-1^ soil), the proportions of Pb in various chemical forms significantly differed between non-inoculated and inoculated seedlings. For example, inoculated seedlings showed significantly lower proportions (13–55%) of water-soluble Pb (including *F*_d-H_2_O_ and *F*_Ethanol_) and significantly higher proportions (16–38%) of HOAc-exactable Pb (*F*_HOAc_) in the roots compared to non-inoculated seedlings.

Compared to controls, the lowest Pb level (90 mg Pb kg^-1^ soil) resulted in a significant increase in the proportion of Pb in *F*_d-H_2_O_ fraction in all analyzed tissues of the non-inoculated seedlings, whereas that of inoculated seedlings were significantly decreased. At higher Pb levels, the seedlings showed a significant reduction in the proportion of water-soluble Pb and a significant increase in the proportion of inactive Pb forms (including *F*_NaCl_, *F*_HOAc_, *F*_HCl_, and *F*_Residue_) in all tissues tested, irrespective of AMF inoculation. Additionally, inoculated seedlings retained significantly lower proportions of water-soluble Pb along with significantly higher proportions of inactive Pb forms compared to non-inoculated seedlings at 900 and 3000 mg Pb kg^-1^ soil (e.g., 21 and 79% versus 28 and 72%, respectively, at 900 mg Pb kg^-1^ soil, respectively; **Figure 6**).

### Two-Factorial ANOVA and Correlation Analysis

The proportions of Pb content in different subcellular fractions and chemical forms were generally correlated with the relative differences in plant growth and physiological parameters of *R. pseudoacacia* seedlings (Supplementary Tables S1–S4). Additionally, Pb, AMF, and their interactions showed significant effects on plant growth and photosynthetic parameters (Supplementary Table S5).

## Discussion

Pb has become ubiquitous in the soil due to natural deposits and intensive human activities (Fahr et al., 2013). AMF could offer an attractive system to facilitate plant-based environmental clean-up and strengthen plant tolerance to HM (Göhre and Paszkowski, 2006). In this study, we cultivated Pb-resistant *R. pseudoacacia* with *F. mosseae* exposed to different Pb levels in a pot experiment, in order to investigate the growth performance of *R. pseudoacacia* plants and Pb accumulation patterns in different tissues of *R. pseudoacacia*. The results indicate the potential of *R. pseudoacacia* for accumulation of Pb in phytoremediation and highlights the binding of inactive Pb forms to cell walls as a mechanism of Pb tolerance in plants with AMF.

### Effects of AMF Symbiosis under Pb Stress

It is well documented that a symbiosis with AMF can improve photosynthesis, water use efficiency, and growth of plants (Chen et al., 2005; Zhu et al., 2011). In the current study, a symbiosis with *F. mosseae* showed significant positive effects on the gas exchange parameters, efficiency of PSII photochemistry, and plant growth in *R. pseudoacacia* seedlings under control conditions (0 mg Pb kg^-1^ soil; **Figures 3**, **4**). This may be attributed to AMF regulation of the availability and uptake of water and nutrients by decreasing stomatal resistance, and increasing CO_2_ assimilation, and accelerating transpiration fluxes (Zhu et al., 2011). Low levels of HM (e.g., 50 μg L^-1^ Cu and Cd) have been shown to exert a stimulatory effect on plant growth of mycorrhizal (*Acaulospora laevis* and *Glomus caledonium*) and non-mycorrhizal *Z. mays* (Liao et al., 2003). Similar to these findings, across all Pb levels investigated in the present study, the lowest Pb level (90 mg Pb kg^-1^ soil) resulted in an increase in plant growth (e.g., higher total dry weight, plant height, and stem diameter; **Figure 2**) coinciding with an improvement of photosynthesis (e.g., higher A, gsw, E, F_v_/F_m_, and ΦPSII; **Figures 3**, **4**) in *R. pseudoacacia* seedlings, regardless of mycorrhization with *F. mosseae*. This growth promotion effect of low Pb may be associated with stimulated metabolism (e.g., photosynthesis) and enzyme activities (e.g., superoxide dismutase and peroxidase) under low Pb stress (Schützendübel and Polle, 2002). Additionally, we found that seedlings inoculated with *F. mosseae* gained a higher performance in growth and photosynthesis than non-inoculated seedlings, consistent with previous findings in other plants. Chen et al. (2007) highlighted the importance of *F. mosseae* in accelerating growth of *Coreopsis drummondii*, *Pteris vittata*, and *Trifolium repens* in Cu mine tailings.

In this study, the application of higher Pb levels (900 and 3000 mg Pb kg^-1^ soil) resulted in an inhibition of photosynthesis, along with a decline in plant growth. The photosynthetic parameters of inoculated and non-inoculated seedlings exhibited similar patterns in response to Pb application; but the effect of AMF inoculation on photosynthetic parameters was strengthened at relatively low Pb level. In stressful conditions with HM, component disruption of the photosynthetic apparatus can occur and photosynthetic processes would be negatively affected in plants (Powles, 1984). Because of a significant reduction in ETR and ΦPSII in the leaves of *R. pseudoacacia* (**Figure 4** and Supplementary Figure S1), it is assumed that higher Pb could destroy the PSII reaction center or disrupt electron transport in the photosynthetic apparatus. Nonetheless, we found that the photosynthetic parameters of inoculated seedlings were less impaired during exposure to higher Pb levels compared to non-inoculated seedlings. For instance, the relative difference in F_v_/F_m_ was significantly lower for leaves of the inoculated seedlings compared to non-inoculated controls for the same Pb treatments (900 or 3000 mg Pb kg^-1^ soil). The higher performance of inoculated plants exposed to high Pb can be related to their improved availability of water and nutrients due to the AMF symbiosis. As we have normalized the physiological parameters of plants grown under Pb exposure to those under control conditions, the higher performance of inoculated plants under high Pb stress may also be attributed to other mechanisms that resist Pb uptake from soil and/or tolerate Pb within the cell (Wang Y. et al., 2015). AMF could facilitate Pb retention within the roots and stems, while reducing Pb accumulation in the leaves (**Table 2**). Thus, although significant Pb accumulation resulted in photosynthesis stress, it was seemed to be diminished for inoculated plants.

In this study, Pb accumulated in the roots, stems and leaves of *R. pseudoacacia* in a concentration-dependent manner, and Pb retention in the roots was significantly higher for inoculated seedlings compared to non-inoculated plants over the entire range of Pb levels applied (**Table 2**). AMF shows high tolerance to HM (Xu et al., 2012) and AMF symbiosis could create a more balanced environment for the roots by enriching HM at or in the mycorrhizal structure, decreasing free ion activity, and reducing toxicity (Chen et al., 2007). During symbiotic interaction between AMF and plants, hyphal network may functionally extend the root system of their hosts to take up HM from an enlarged soil volume (Göhre and Paszkowski, 2006). This explains the twofold higher Pb content in the roots of inoculated seedlings compared to non-inoculated seedlings of *R. pseudoacacia* (**Table 2**). The mitigation of negative impacts induced by HM could vary to a large extent, depending on HM species, soil HM level, fungal symbiotic partner, and/or plant environment (Hildebrandt et al., 2007). In the present study, the colonization of *F. mosseae* was markedly reduced in the roots of *R. pseudoacacia* at 900 and 3000 mg Pb kg^-1^ soil (**Figure 1**), indicating the negative effect of high Pb on the root colonization of AMF. A similar phenomenon was found by Weissenhorn and Leyval (1995) in the roots of *Z. mays* under Cd stress (up to 10 mg L^-1^), maybe due to limited hyphal extension and restricted spore germination at high HM levels (Weissenhorn et al., 1994). It is noteworthy that the root colonization rate by *F. mosseae* was not completely eliminated at 3000 mg Pb kg^-1^ soil in the current study. Thus, we assume that *F. mosseae* possesses a high tolerance toward Pb and it maybe a candidate for applications in reclamation of Pb polluted soils. This assumption needs to be tested by further long-term experiments.

### Molecular Mechanism of Pb Tolerance

Under Pb stress, some molecular mechanisms are rapidly activated to minimize the potential toxicity of HM in plants (Wang Y. et al., 2015). Compartmentalization of HM is an important mechanism of detoxification/adaptation in plant cells (Zhao et al., 2015). After entering the cells, HM are bound to various subcellular compartments and exhibit different ecotoxicological significances. The cell walls, as the first protecting barrier, are mainly composed of polyoses (including cellulose, hemicellulose, and pectin) and proteins (Wang Y. et al., 2015). The negatively charged sites provided by functional groups, such as hydroxyl, carboxyl, amino, and aldehyde groups (Hu et al., 2012), can bind HM ions and limit their transport across the cell membrane (Bhatia et al., 2005; Arias et al., 2010). In the present study, a large proportion of Pb was found in the cell wall fraction in the roots, stems, and leaves of *R. pseudoacacia* seedlings across all Pb treatments, irrespective of AMF inoculation (**Figure 5**). This observation leads to the conclusion that the cell walls function as the primary barrier to Pb entry into the cytoplasm and limit organelles from suffering Pb toxicity in *R. pseudoacacia*. Further research is needed to evaluate the contribution of Pb sequestration in the cell walls of *R. pseudoacacia* roots.

The cell matrix between the cell walls and organelles can be regarded as an intracellular buffer (Arias et al., 2010). Soluble cellular components store HM, while they contain organo-ligands, mainly sulfur-rich peptides, organic alkali, and organic acids. Complexing metals with organo-ligands in these storage sites can decrease free ion activity and reduce HM toxicity (Bhatia et al., 2005). In the current study, the proportion of Pb in the soluble fraction was significantly increased in different tissues of *R. pseudoacacia* seedlings with increasing Pb level (**Figure 5**). This supports the hypothesis that complex formation of metals with organo-ligands is a molecular mechanism reducing HM toxicity in *R. pseudoacacia* seedlings. Meanwhile, the proportion of Pb in different subcellular fractions was significantly affected by AMF inoculation (Supplementary Table S5). Compared to the non-inoculated seedlings, *R. pseudoacacia* seedlings with *F. mosseae* showed significantly higher proportions of Pb in the cell wall and soluble fractions in the roots, stems, and leaves of *R. pseudoacacia* seedlings over the entire range of Pb level applied (**Figure 5**). This indicates that AMF facilitated the immobilization of Pb in the cell wall and soluble fractions of plant tissues, similar to the finding in *Medicago sativa* with *Glomus intraradices* under Cd stress (Wang et al., 2011). Moreover, the proportion of Pb in the subcellular compartments was highly associated with photosynthesis and plant growth of *R. pseudoacacia* seedlings (Supplementary Tables S1, S2). Thus, we conclude that the selective distribution of Pb in the cell wall and soluble fractions is a strategy for Pb tolerance and detoxification during the growth of *R. pseudoacacia* seedlings with AMF.

A high level of HM in plant tissue does not necessarily mean a high toxicity for a plant, since the HM may exist in chemical forms with low or no phytotoxicity (Fu et al., 2011). In the present study, we found that inoculation with *F. mosseae* promoted the conversion of Pb into inactivate forms (*F*_NaCl_, *F*_HOAc_, *F*_HCl_, and *F*_Residue_; **Figure 6**). The undissolved phosphate (*F*_HOAc_) and oxalate (*F*_HCl_) fractions of Pb have been already described as effective means in Pb detoxification (Zhang et al., 2013; Wang Y. et al., 2015) as they are less harmful than soluble Pb to plant cells. Wang et al. (2016) have indicated that the fibrous roots of apple tree showed largest proportions in the HOAc and HCl extractable Cu forms. Similarly, Zhao et al. (2015) showed that NaCl- and HOAc-extractable HM may be responsible for the adaptation of *Porphyra yezoensis* to Cd stress.

According to the high abundance of inactive forms of Pb in different tissues of inoculated plants, we therefore assume that (i) AMF may have a significant impact on the detoxification of Pb in plants through the transformation of Pb into the inactive forms, and (ii) transformation of Pb in inoculated seedlings may base on chelation of Pb by specific polar materials (e.g., hydroxyl or carboxyl groups) to form a non-toxic complex (Andrade et al., 2010), thereby contributing to improved plant growth and physiological performance. During exposure to high Pb, better photosynthesis and performance of PSII in the leaves of inoculated plants (e.g., higher gsw and F_v_/F_m_) may be attributed to the retention of Pb in different subcellular fractions and chemical forms in the roots, which could prevent the disruption of photosynthesis apparatus and membrane integrity. AMF symbiosis could improve the capacity of gas exchange, the efficiency of photochemistry and non-photochemistry of PSII, and regulate the energy bifurcation between photochemical and non-photochemical events in the leaves of seedlings.

Given its high stress tolerance and fast growth, *R. pseudoacacia* is considered suitable for soil and vegetation restoration in HM contaminated areas (Yang et al., 2015b,c). In this study, the majority of Pb was found to be retained in the roots of *R. pseudoacacia* under Pb stress, with a significantly higher accumulation in seedlings with *F. mosseae*. Compared with non-inoculated seedlings, the improved physiological parameters were highly associated with Pb compartmentalization in different chemical forms, including (1) increased proportion of Pb in the cell wall and soluble fractions, with the highest proportion of Pb in the cell wall fraction; and (2) increased proportion of inactive Pb, especially *F*_NaCl_ and *F*_HOAc_, in plant tissues. These provide new insights into the role of AMF on Pb tolerance in woody legumes from a molecular perspective. From an ecological point of view, *R. pseudoacacia* inoculated with *F. mosseae* may be used for remediating Pb polluted soils.

## Author Contributions

All authors participated in the conception of the topic after critically revising it for important intellectual content. LH and HZ wrote the manuscript. YS, YY, and HC assisted with data analysis, manuscript preparation, and revision. MT served as the primary investigator, conceived the project, and finalized the manuscript. All authors read and approved the final manuscript.

## Conflict of Interest Statement

The authors declare that the research was conducted in the absence of any commercial or financial relationships that could be construed as a potential conflict of interest.
