# Supplementary material for: Subcellular Compartmentalization and Chemical Forms of Lead Participate in Lead Tolerance of Robinia pseudoacacia L. with Funneliformis mosseae
Source: Front Plant Sci. 2017 Apr 10;8:517. doi: 10.3389/fpls.2017.00517 (PMC5385381; doi:10.3389/fpls.2017.00517)
Supplement: Supplementary file 1 [file Presentation_1.pdf]

SUPPLEMENTAL MATERIALS

Subcellular Compartmentalization and Chemical Forms of Lead Participate in Lead Tolerance of *Robinia pseudoacacia* L. with *Funneliformis mosseae*

Li Huang* [1]*, Haoqiang Zhang* [2]*, Yingying Song* [1]*, Yurong Yang* [2]*,
Hui Chen* [2] *and Ming Tang* [2]*

*Correspondence: Ming Tang

Email: tangm@nwsuaf.edu.cn

Table S1 Correlation analysis between the proportions of Pb in different subcellular fractions and the relative differences in plant growth parameters of *Robinia pseudoacacia* seedlings in response to 0, 90, 900, and 3000 mg Pb kg^{-1} soil for four months.

Plant tissue	Subcellular fraction of Pb	Root dry weight	Stem dry weight	Leaf dry weight	Total biomass	Plant height	Stem diameter
Root	FI (cell wall)	-0.553^{**}	-0.562^{**}	-0.670^{**}	-0.613^{**}	-0.580^{**}	-0.526^{**}
	FII (organelle)	0.645^{**}	0.628^{**}	0.741^{**}	0.694^{**}	0.659^{**}	0.613^{**}
	FIII (soluble)	-0.706^{**}	-0.671^{**}	-0.785^{**}	-0.747^{**}	-0.711^{**}	-0.671^{**}
Stem	FI (cell wall)	-0.642^{**}	-0.668^{**}	-0.730^{**}	-0.693^{**}	-0.671^{**}	-0.624^{**}
	FII (organelle)	0.517^{**}	0.561^{**}	0.605^{**}	0.568^{**}	0.550^{**}	0.500^{*}
	FIII (soluble)	-0.162^{NS}	-0.240^{NS}	-0.235^{NS}	-0.205^{NS}	-0.199^{NS}	-0.152^{NS}
Leaf	FI (cell wall)	-0.639^{**}	-0.635^{**}	-0.748^{**}	-0.696^{**}	-0.662^{**}	-0.612^{**}
	FII (organelle)	0.661^{**}	0.644^{**}	0.757^{**}	0.711^{**}	0.676^{**}	0.629^{**}
	FIII (soluble)	-0.677^{**}	-0.649^{**}	-0.762^{**}	-0.721^{**}	-0.684^{**}	-0.640^{**}

Pearson correlation coefficients *r* were determined across all Pb exposure experiments (0, 90, 900, 3000 mg Pb kg^{-1} soil) ($n = 48$). Asterisks indicate significant differences (* $P < 0.05$; ** $P < 0.01$). NS = not significant.

Table S2 Correlation analysis between the proportion of Pb in different subcellular fractions and the relative differences in physiological parameters of *Robinia pseudoacacia* seedlings in response to 0, 90, 900, and 3000 mg Pb kg^{-1} soil for four months.

Plant tissue	Subcellular fraction of Pb	F_v/F_m	ΦPSII	F_v/F_o	ETR	qP	qN	A	gsw	C_i	E
Root	FI (cell wall)	−0.641**	−0.668**	−0.486*	−0.629**	−0.660**	0.465*	−0.585**	−0.460*	0.548**	−0.611**
	FII (organelle)	0.706**	0.737**	0.586**	0.704**	0.727**	−0.559**	0.657**	0.551**	−0.628**	0.673**
	FIII (soluble)	−0.747**	−0.779**	−0.654**	−0.753**	−0.768**	0.623**	−0.703**	−0.613**	0.681**	−0.711**
Stem	FI (cell wall)	−0.731**	−0.736**	−0.578**	−0.710**	−0.746**	0.583**	−0.668**	−0.547**	0.622**	−0.690**
	FII (organelle)	0.620**	0.615**	0.451*	0.589**	0.634**	−0.471*	0.545**	0.412*	−0.491*	0.572**
	FIII (soluble)	−0.280^{NS}	−0.252^{NS}	−0.102^{NS}	−0.233^{NS}	−0.287^{NS}	0.151^{NS}	−0.190^{NS}	−0.052^{NS}	0.127^{NS}	−0.224^{NS}
Leaf	FI (cell wall)	−0.715**	−0.746**	−0.577**	−0.709**	−0.734**	0.550**	−0.666**	−0.551**	0.633**	−0.685**
	FII (organelle)	0.724**	0.754**	0.603**	0.722**	0.745**	−0.575**	0.673**	0.565**	−0.641**	0.687**
	FIII (soluble)	−0.729**	−0.759**	−0.622**	−0.731**	−0.751**	0.594**	−0.676**	−0.574**	0.646**	−0.686**

Pearson correlation coefficients *r* were determined across all Pb exposure experiments (0, 90, 900, 3000 mg Pb kg^{-1} soil) (n = 48).
Asterisks indicate significant differences (* $P < 0.05$; ** $P < 0.01$). NS = not significant.

Table S3 Correlation analysis between the proportions of Pb in different chemical forms and the relative difference in plant growth parameters of *Robinia pseudoacacia* seedlings in response to 0, 90, 900, and 3000 mg Pb kg^{-1} soil for four months.

Plant tissue	Chemical form of Pb	Root dry weight	Stem dry weight	Leaf dry weight	Total biomass	Plant height	Stem diameter
Root	$F_{Ethanol}$	0.584^{**}	0.676^{**}	0.715^{**}	0.662^{**}	0.654^{**}	0.602^{**}
	F_{H2O}	0.788^{**}	0.751^{**}	0.840^{**}	0.817^{**}	0.790^{**}	0.761^{**}
	F_{NaCl}	-0.571^{**}	-0.580^{**}	-0.556^{**}	-0.572^{**}	-0.569^{**}	-0.552^{**}
	F_{HOAc}	-0.672^{**}	-0.675^{**}	-0.776^{**}	-0.728^{**}	-0.701^{**}	-0.659^{**}
	F_{HCl}	-0.829^{**}	-0.841^{**}	-0.865^{**}	-0.857^{**}	-0.849^{**}	-0.822^{**}
	$F_{Residue}$	-0.854^{**}	-0.787^{**}	-0.915^{**}	-0.885^{**}	-0.848^{**}	-0.819^{**}
Stem	$F_{Ethanol}$	0.743^{**}	0.749^{**}	0.868^{**}	0.810^{**}	0.782^{**}	0.735^{**}
	F_{H2O}	0.782^{**}	0.834^{**}	0.836^{**}	0.823^{**}	0.820^{**}	0.781^{**}
	F_{NaCl}	-0.807^{**}	-0.894^{**}	-0.850^{**}	-0.847^{**}	-0.858^{**}	-0.824^{**}
	F_{HOAc}	-0.690^{**}	-0.685^{**}	-0.776^{**}	-0.737^{**}	-0.710^{**}	-0.667^{**}
	F_{HCl}	-0.358^{NS}	-0.200^{NS}	-0.452^{*}	-0.387^{NS}	-0.317^{NS}	-0.291^{NS}
	$F_{Residue}$	-0.667^{**}	-0.692^{**}	-0.817^{**}	-0.747^{**}	-0.720^{**}	-0.666^{**}
Leaf	$F_{Ethanol}$	0.572^{**}	0.624^{**}	0.735^{**}	0.659^{**}	0.634^{**}	0.574^{**}
	F_{H2O}	0.813^{**}	0.845^{**}	0.867^{**}	0.852^{**}	0.843^{**}	0.807^{**}
	F_{NaCl}	-0.813^{**}	-0.875^{**}	-0.864^{**}	-0.854^{**}	-0.856^{**}	-0.820^{**}
	F_{HOAc}	-0.715^{**}	-0.711^{**}	-0.823^{**}	-0.773^{**}	-0.743^{**}	-0.698^{**}
	F_{HCl}	-0.388^{NS}	-0.403^{NS}	-0.478^{*}	-0.436^{*}	-0.416^{*}	-0.380^{NS}
	$F_{Residue}$	-0.575^{**}	-0.606^{**}	-0.720^{**}	-0.652^{**}	-0.621^{**}	-0.562^{**}

Pearson correlation coefficients *r* were determined across all Pb exposure experiments (0, 90, 900, 3000 mg Pb kg^{-1} soil) (n = 48). Asterisks indicate significant differences (* $P < 0.05$; ** $P < 0.01$). NS = not significant.

Table S4 Correlation analysis between the proportions of Pb in different chemical forms and the relative differences in physiological parameters of *Robinia pseudoacacia* seedlings in response to 0, 90, 900, and 3000 mg Pb kg^{-1} soil for four months.

Plant tissue	Chemical forms of Pb	F_v/F_m	ΦPSII	F_v/F_o	ETR	qP	qN	A	gsw	C_i	E
Root	$F_{Ethanol}$	0.708**	0.722**	0.499*	0.668**	0.717**	−0.508*	0.691**	0.580**	−0.648**	0.743**
	F_{H2O}	0.805**	0.834**	0.739**	0.813**	0.827**	−0.714**	0.782**	0.724**	−0.761**	0.788**
	F_{NaCl}	−0.587**	−0.560**	−0.517**	−0.564**	−0.603**	0.575**	−0.531**	−0.475*	0.504*	−0.547**
	F_{HOAc}	−0.731**	−0.771**	−0.610**	−0.728**	−0.751**	0.578**	−0.719**	−0.640**	0.692**	−0.744**
	F_{HCl}	−0.869**	−0.870**	−0.773**	−0.855**	−0.884**	0.787**	−0.842**	−0.779**	0.811**	−0.856**
	$F_{Residue}$	−0.864**	−0.906**	−0.817**	−0.887**	−0.884**	0.764**	−0.842**	−0.783**	0.832**	−0.833**
Stem	$F_{Ethanol}$	0.822**	0.866**	0.687**	0.821**	0.835**	−0.638**	0.810**	0.717**	−0.786**	0.828**
	F_{H2O}	0.874**	0.853**	0.721**	0.840**	0.880**	−0.755**	0.813**	0.702**	−0.764**	0.831**
	F_{NaCl}	−0.906**	−0.869**	−0.737**	−0.858**	−0.908**	0.799**	−0.855**	−0.754**	0.809**	−0.885**
	F_{HOAc}	−0.762**	−0.783**	−0.645**	−0.759**	−0.779**	0.619**	−0.710**	−0.603**	0.661**	−0.715**
	F_{HCl}	−0.329^{NS}	−0.426*	−0.370^{NS}	−0.400^{NS}	−0.350^{NS}	0.225^{NS}	−0.320^{NS}	−0.282^{NS}	0.343^{NS}	−0.283^{NS}
	$F_{Residue}$	−0.768**	−0.816**	−0.606**	−0.762**	−0.778**	0.552**	−0.760**	−0.656**	0.735**	−0.786**
Leaf	$F_{Ethanol}$	0.694**	0.735**	0.497*	0.674**	0.703**	−0.460*	0.677**	0.556**	−0.648**	0.718**
	F_{H2O}	0.888**	0.877**	0.754**	0.864**	0.896**	−0.776**	0.836**	0.736**	−0.800**	0.851**
	F_{NaCl}	−0.901**	−0.878**	−0.746**	−0.864**	−0.905**	0.789**	−0.853**	−0.754**	0.817**	−0.878**
	F_{HOAc}	−0.785**	−0.821**	−0.659**	−0.784**	−0.800**	0.621**	−0.758**	−0.661**	0.734**	−0.773**
	F_{HCl}	−0.462*	−0.482*	−0.354^{NS}	−0.455*	−0.469*	0.327^{NS}	−0.431*	−0.345^{NS}	0.402^{NS}	−0.442*
	$F_{Residue}$	−0.687**	−0.722**	−0.506*	−0.672**	−0.701**	0.473*	−0.646**	−0.514*	0.608**	−0.677**

Pearson correlation coefficients *r* were determined across all Pb exposure experiments (0, 90, 900, 3000 mg Pb kg^{-1} soil) ($n = 48$). Asterisks indicate significant differences (* $P < 0.05$; ** $P < 0.01$). NS = not significant.

Table S5 Effects of Pb treatments (Pb), AMF treatments (AMF) and Pb × AMF on the parameters of *Robinia pseudoacacia* seedlings in response to 0, 90, 900, and 3000 mg Pb kg^{-1} soil for four months.

Parameters	Pb	AMF	Pb × AMF
Percent Pb in FI (cell wall) of roots	**	**	**
Percent Pb in FII (organelle) of roots	**	**	**
Percent Pb in FIII (soluble) of roots	**	**	**
Percent Pb in FI (cell wall) of stems	**	**	**
Percent Pb in FII (organelle) of stems	**	**	**
Percent Pb in FIII (soluble) of stems	**	**	**
Percent Pb in FI (cell wall) of leaves	**	**	**
Percent Pb in FII (organelle) of leaves	**	**	**
Percent Pb in FIII (soluble) of leaves	**	**	**
Percent Pb in $F_{Ethanol}$ in roots	**	**	**
Percent Pb in F_{H2O} in roots	**	*	**
Percent Pb in F_{NaCl} in roots	**	**	**
Percent Pb in F_{HOAc} in roots	**	**	**
Percent Pb in F_{HCl} in roots	**	**	**
Percent Pb in $F_{Residue}$ in roots	**	**	**
Percent Pb in $F_{Ethanol}$ in stems	**	**	NS
Percent Pb in F_{H2O} in stems	**	**	**
Percent Pb in F_{NaCl} in stems	**	**	**
Percent Pb in F_{HOAc} in stems	**	**	**
Percent Pb in F_{HCl} in stems	**	**	**
Percent Pb in $F_{Residue}$ in stems	**	**	**
Percent Pb in $F_{Ethanol}$ in leaves	**	**	**
Percent Pb in F_{H2O} in leaves	**	**	**
Percent Pb in F_{NaCl} in leaves	**	**	**
Percent Pb in F_{HOAc} in leaves	**	**	**
Percent Pb in F_{HCl} in leaves	**	**	**
Percent Pb in $F_{Residue}$ in leaves	**	**	**
F_v/F_m	**	**	*
ΦPSII	**	**	**
F_v/F_o	**	**	**
ETR	**	**	*
qP	**	**	**
qN	**	**	*
A	**	**	**
gsw	**	**	**
C_i	**	**	**
E	**	**	**
Root dry weight	**	**	**
Stem dry weight	**	**	**
Leaf dry weight	**	**	**

Total biomass	**	**	**
Plant height	**	**	**
Stem diameter	**	**	**

A two-factorial ANOVA was performed across all Pb exposure experiments (0, 90, 900, 3000 mg Pb kg^{-1} soil) (n = 48). Asterisks indicate significant differences (* $P < 0.05$; ** $P < 0.01$). NS = not significant.

FIGURE LEGENDS:

Figure S1 Relative differences in kinetic parameters of chlorophyll fluorescence in leaves of *Robinia pseudoacacia* seedlings with (+M) or without (−M) *Funneliformis mosseae* in response to 0, 90, 900, and 3000 mg Pb kg^{-1} soil for four months. (A) Potential activity of PSII, F_v/F_o; (B) Electron transport rate, ETR; (C) Photochemical quenching coefficient, qP; and (D) Non-photochemical quenching values, qN.

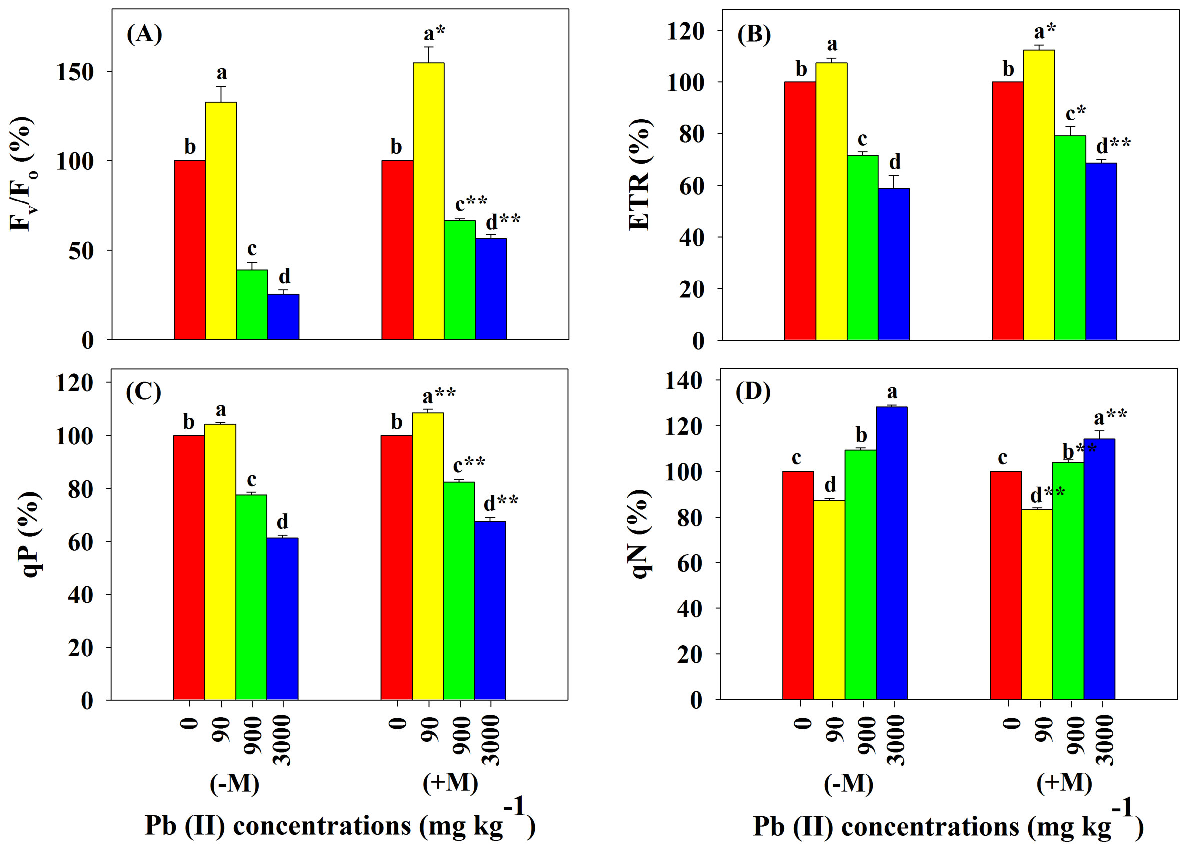

Figure S1 Relative differences in kinetic parameters of chlorophyll fluorescence in leaves of *Robinia pseudoacacia* seedlings with (+M) or without (−M) *Funneliformis mosseae* in response to 0, 90, 900, and 3000 mg Pb kg^{-1} soil for four months. (A) Potential activity of PSII, F_v/F_o; (B) Electron transport rate, ETR; (C) Photochemical quenching coefficient, qP; and (D) Non-photochemical quenching values, qN. Shown are means ± SD (n = 6). Asterisks indicate significant differences between inoculated and non-inoculated seedlings within one Pb level (* $P < 0.05$, ** $P < 0.01$; t-test). Different lower case letters indicate significant differences among different Pb levels within inoculated or non-inoculated treatment ($P < 0.05$; ANOVA with *post hoc* Duncan).
